# Zinc Permeation Through Acid-Sensing Ion Channels

**DOI:** 10.3390/cells15020186

**Published:** 2026-01-20

**Authors:** Xiang-Ping Chu, Koichi Inoue, Zhi-Gang Xiong

**Affiliations:** 1 Department of Biomedical Sciences, School of Medicine, University of Missouri-Kansas City, Kansas City, MO 64108, USA; 2 Department of Neurobiology, Neuroscience Institute, Morehouse School of Medicine, Atlanta, GA 30310, USA; zxiong@msm.edu; 3 Department of Anatomy and Cell Biology, Nara Medical University, Kashihara 634-8521, Nara, Japan

**Keywords:** zinc, ASIC, acidosis, stroke, neurotoxicity

## Abstract

**Highlights:**

**What are the main findings?**

- Acid-sensing ion channel 2a (ASIC2a)-containing channels are permeable to zinc.
- Zinc enhances neuronal injury under severe acidic conditions.

**What is the implication of the main findings?**

- ASIC2a may play a role in zinc toxicity under low pH conditions such as in severe ischemia.

**Abstract:**

Acid-sensing ion channels (ASICs), activated under acidic conditions, play a critical role in ischemic brain injury, but the detailed mechanisms and signaling pathways remain unclear. Our previous studies have shown that activation of ASIC1a channels contributes to acidosis-induced neuronal injury, partially mediated by increased calcium influx. In this study, we provide evidence that activation of ASIC2a-containing channels induces zinc influx. In cultured mouse cortical neurons, ASIC currents that were insensitive to PcTx1 inhibition were potentiated by extracellular zinc. In Chinese Hamster Ovary cells transfected with different ASIC subunits, large inward currents were recorded upon a pH drop from 7.4 to 5.0 in cells expressing homomeric ASIC1a, ASIC2a, or heteromeric ASIC1a/2a channels when normal Na^+^-rich extracellular fluid (ECF) was used. However, when ECF was modified to one containing zinc as the primary cation, the same pH drop induced an inward current only in cells expressing homomeric ASIC2a or heteromeric ASIC1a/2a, but not homomeric ASIC1a. Fluorescence imaging revealed rapid zinc influx in cells expressing ASIC2a but not ASIC1a when zinc was applied with the acidic ECF. Additionally, at pH values where ASIC2a-containing channels were activated, acid-mediated neurotoxicity was exacerbated by zinc. Thus, ASIC2a-containing channels may represent a novel pathway for zinc entry and activation of these channels might contribute to zinc-mediated neurotoxicity.

## 1. Introduction

Stroke ranks among the leading causes of death worldwide [1,2]. Even if it is not fatal, it often results in severe physical and mental disabilities, requiring long-term rehabilitation. Therefore, understanding the mechanisms of stroke-mediated brain injury and developing better treatment strategies are major challenges in the field.

In stroke, whether it is ischemic or hemorrhagic, an occlusion or a rupture of cerebral blood vessels occurs, which leads to impaired blood flow. In both cases, neuronal cells suffer the most from resulting insufficiency in blood and oxygen supply. In the case of ischemic stroke, accumulation of glutamate due to increased release from the presynaptic terminals and glial cells results in excessive Ca^2+^ entry through N-methyl-D-aspartate (NMDA) receptors and subsequent neuronal injury [3,4]. This Ca^2+^ accumulation in neurons was considered the main factor in neuronal damage during ischemic stroke. However, Stork and Li reported that the previously assumed increase in Ca^2+^ during ischemia and reperfusion was at least partially due to an increase in zinc [5]. In fact, zinc-mediated neurotoxicity in cerebral ischemia has long been reported by Koh et al. [6]. It was shown that the zinc toxicity was mediated by NMDA receptors on the postsynaptic membrane, which have been reported to be permeable to zinc [7,8]. Additionally, α-amino-3-hydroxy-5-methyl-4-isoxazolepropionic acid (AMPA) receptors and voltage-dependent calcium channels (VDCCs) have also been reported to be zinc-permeable [9,10], suggesting that they may mediate zinc damage to neurons in addition to Ca^2+^ toxicity. Accordingly, blocking these receptors/channels has been shown to suppress ischemic neuronal cell death. However, in prolonged ischemia, inhibiting glutamate receptors and VDCCs was insufficient to block the increase in intracellular Ca^2+^ and neuronal cell death [11]. It was found that this glutamate receptors- and VDCCs-independent neuronal cell death was partially mediated by the activation of transient receptor potential melastatin 7 (TRPM7) [11], and that zinc permeation played some roles [12]. Thus, it has become clear that, in addition to the Ca^2+^ toxicity, zinc influx through several ion channels can also promote neuronal damage. However, it is unclear whether other ion channels in neurons also mediate zinc permeation and potentially participate in brain damage during ischemia and other neurological conditions.

Acid-sensing ion channels (ASICs) are proton-gated cation channels belonging to epithelial sodium channels/degenerin superfamily [13]. In addition to Na^+^, homomeric ASIC1a channels, but not homomeric ASIC2a or heteromeric ASIC1a/2a channels, are permeable to Ca^2+^ and Ca^2+^ influx through these channels contributes to neuronal damage in brain ischemia [14,15]. Although less studied for the pathological functions due to a low sensitivity to H^+^, ASIC2a has also been shown to play various physiological roles such as mechanosensation, synaptic targeting, and cardiovascular responses [16,17]. Here, we show that ASIC2a-containing channels are also permeable to zinc and that activation of these channels likely contributes to zinc-mediated neuronal damage in ischemia.

## 2. Materials and Methods

### 2.1. Animals

Pregnant Swiss mice were purchased from Charles River. Experiments were conducted in accordance with the Guidelines of Institutional Animal Care and Use Committee of Morehouse School of Medicine and University of Missouri-Kansas City.

### 2.2. Cell Culture

Mouse cortical neurons were cultured following established protocols [12,14]. In brief, ~10 pregnant Swiss mice at embryonic day 16 were anesthetized using halothane and euthanized by cervical dislocation. Fetal brains were quickly extracted and transferred to ice-cold, calcium- and magnesium-free phosphate-buffered saline (PBS). Cerebral cortices were dissected under a dissection microscope and incubated with 0.05% trypsin-EDTA for 10 min at 37 °C, followed by trituration with fire-polished glass pipettes. Cells were counted and plated in poly-L-ornithine-coated culture dishes or 24-well plates at a density of 1 × 10^6^ cells per dish or 2 × 10^5^ cells per well, respectively. Neurons were cultured with Neurobasal medium (Thermo Fisher Scientific, Waltham, MA, USA) supplemented with B-27 (Thermo Fisher Scientific) and glutamine and maintained at 37 °C in a humidified 5% CO_2_ atmosphere incubator. Cultures were fed twice a week. Neurons were used for the experiments between 11 and 15 days in vitro.

Chinese Hamster Ovary (CHO) and Human Embryonic Kidney 293 (HEK293) cells were cultured in F12 and Dulbecco’s modified Eagle’s medium, respectively, supplemented with 10% fetal bovine serum and antibiotics.

### 2.3. Plasmid Construction and Transfection

Plasmids for expression of rat ASIC1a and ASIC2a were kindly provided by Dr. M. Lazdunski. For fluorescence imaging, the open reading frames of rat ASICs were sub-cloned into pIRES-DsRed2 to obtain bicistronic expression of red fluorescent protein, DsRed2 [18,19]. For transfection of the indicated plasmids, FuGENE 6 (Promega, Madison, WI, USA) was used, following the protocol provided by the manufacturer. Transfection efficiency was estimated to be 10–20% according to the co-transfection with eGFP expression plasmid [12]. Then, 2–3 days after transfection, cells were used for experiments.

### 2.4. Solutions

For electrophysiological recordings and zinc imaging, cells were superfused with standard extracellular fluid (ECF) containing (in mM) 140 NaCl, 5.4 KCl, 2 CaCl_2_, 1 MgCl_2_, 10 glucose, and 20 HEPES (pH adjusted to 7.4 with NaOH; osmolarity adjusted to 320–330 mOsm/L using sucrose). For experiments using ECF containing 0.3 mM zinc (Zn-ECF) in Figures 2 and 3, sodium and other permeable cations were replaced with NMDG, a membrane-impermeable cation. In solutions with pH ≤ 6.0, MES was substituted for HEPES to ensure more effective pH buffering [18]. Patch pipettes were filled with an internal solution containing (in mM) 140 CsF, 11 EGTA, 5 tetraethylammonium chloride, and 10 HEPES (pH 7.3, adjusted with CsOH). For cortical neurons, the extracellular solution was supplemented with MK-801 (10 µM), CNQX (20 µM), and nimodipine (5 µM) (all from Sigma-Aldrich, St. Louis, MO, USA) to inhibit potential zinc influx via glutamate receptors and VDCCs, respectively.

### 2.5. Electrophysiology

Whole-cell patch-clamp recordings were conducted according to protocols detailed in our earlier studies [20,21]. Neurons were placed on the stage of an inverted Nikon microscope and continuously superfused with ECF at room temperature. Patch pipettes were pulled from 1.5 mm diameter borosilicate glass capillaries (WPI, Sarasota, FL, USA) using a Narishige PP-83 puller (Narishige, Tokyo, Japan). When filled with the intracellular solution (as previously described), pipette resistance ranged from 2 to 4 MΩ. Membrane currents were recorded using an Axopatch 200B amplifier (Molecular Devices, San Jose, CA, USA), with signals filtered at 2 kHz and digitized at 5 kHz via a Digidata 1322A acquisition system (Molecular Devices). An SF-77B multi-barrel perfusion system (Warner Instruments, Hamden, CT, USA) enabled rapid solution exchange.

### 2.6. Zinc Imaging

Intracellular zinc levels in HEK293 cells or mouse cortical neurons were visualized using the zinc-sensitive fluorescent dye FluoZin-3 (Thermo Fisher Scientific), following procedures outlined in our previous work [12]. Cells cultured on glass coverslips were incubated with 5 µM FluoZin-3-AM in standard ECF for 30 min at 37 °C, followed by a 30 min de-esterification period at room temperature (22–25 °C). Coverslips were then mounted in a recording chamber on the stage of an inverted microscope (Eclipse TE2000-U; Nikon, Tokyo, Japan) and superfused with standard ECF at room temperature for at least 10 min before imaging. FluoZin-3 fluorescence was excited at 490 nm and collected through a 500–550 nm band-pass filter. Images were acquired using a 40 × Super Fluor objective lens (numerical aperture 0.90; Nikon) and a CoolSNAP ES2 CCD camera (Photometrics, Tucson, AZ, USA), with Imaging Workbench 6.0 software (INDEC BioSystems, Los Altos, CA, USA). Changes in fluorescence intensity (Figures 4 and 5) were quantified relative to baseline fluorescence (F), calculated as the average of five initial data points.

### 2.7. Lactate Dehydrogenase (LDH) Assay

Lactate dehydrogenase (LDH) release was measured following protocols described in our previous studies [12]. Cells cultured in 24-well plates were first rinsed with PBS. To assess baseline LDH levels, 50 µL of culture medium was collected from each well and transferred to a 96-well plate. Cells were then exposed to zinc containing ECF at different pH (see Section 2.4) for 1 h, followed by 5 h incubation in normal culture medium. Subsequently, 50 µL of medium was collected from each well for LDH release measurement. To determine the maximum LDH release, cells were treated with 0.5% Triton X-100 (Sigma-Aldrich) for 30 min at the end of the experiment. For all samples, 50 µL of the assay reagent from the Cytotoxicity Detection Kit (Roche Diagnostics, Indianapolis, IN, USA) was added to 50 µL of sample, following the manufacturer’s instructions. After 30 min incubation, absorbance at 492 nm and 620 nm was measured using a spectrophotometer (SpectraMax Plus; Molecular Devices). Final LDH values were calculated by subtracting absorbance at 620 nm from that at 492 nm.

### 2.8. Statistical Analysis

**Figure 1:**
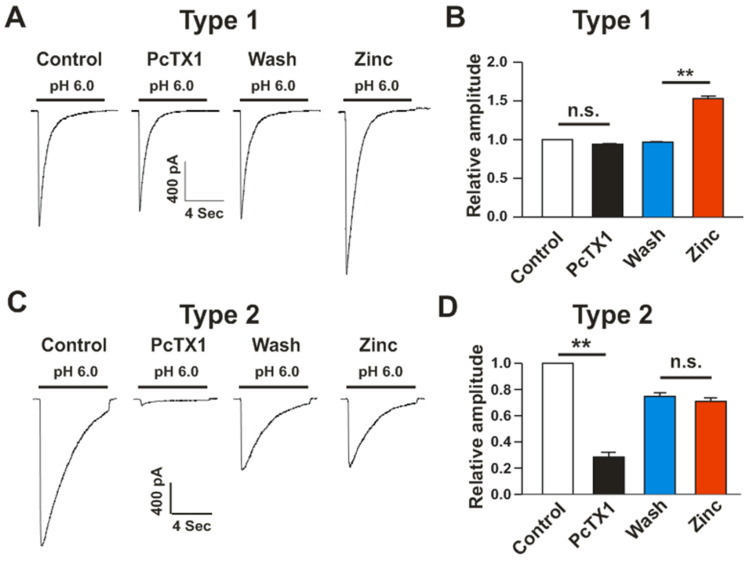
Effects of zinc on PcTx1-senstitive and -insensitive ASIC currents in cultured mouse cortical neurons. Representative traces showing the effects of zinc on PcTx1-insensitive ASIC currents (**A**) and PcTx1-sensitive currents (**C**) in cultured mouse cortical neurons. (**B**) Summary data showing zinc potentiation of the ASIC currents in PcTx1-insensitive cells. (**D**) Summary data showing the lack of zinc potentiation on the ASIC current in PcTx1-sensitive cells. The first three traces and associated statistical bars represent data from neurons treated with ECF only, while the fourth trace and corresponding statistical bar depict data from neurons treated with normal ECF containing Zn. n = 16–23. ** *p* < 0.01 vs. control or wash, Student’s *t*-test. n.s. denotes not significant.

**Figure 2:**
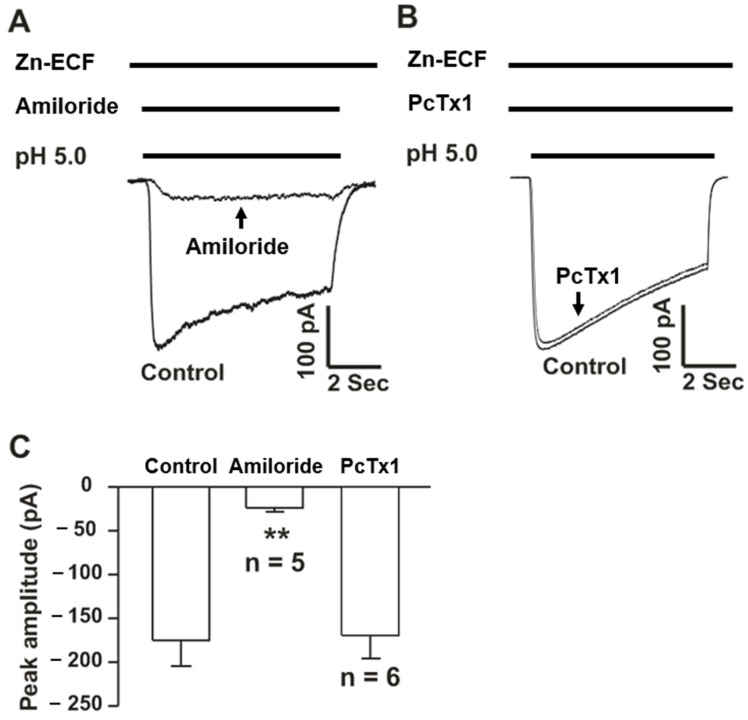
Low pH-induced zinc-mediated currents in cultured mouse cortical neurons. Representative traces showing low pH-induced zinc-mediated current, which was inhibited by amiloride (**A**), but not PcTx1 (**B**). (**C**) Bar graph showing relative changes in the amplitude of zinc-mediated currents by amiloride or PcTx1. All data presented in this figure were obtained from neurons treated exclusively with Zn-ECF. n = 5–6. ** *p* < 0.01 vs. control, one-way ANOVA followed by post hoc Dunnett’s test.

**Figure 3:**
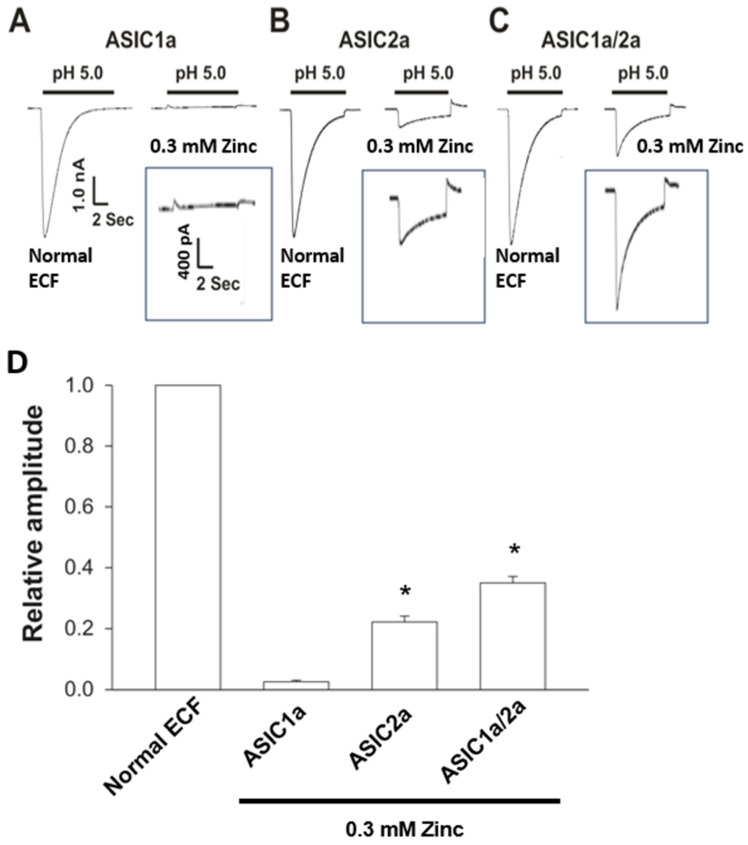
Subunit dependency of zinc-mediated currents. CHO cells were transfected with ASIC1a (**A**), ASIC2a (**B**), or both (**C**). After 2–3 days, patch-clamp recordings were performed. Low pH-induced currents were first recorded in normal ECF, followed by recording in Na^+^, K^+^, and Ca^2+^-free ECF with zinc (0.3 mM) as the only charge carrier. In panels (**A**–**C**), the first traces were obtained from cells treated with normal ECF, whereas the second traces were derived from cells treated with Zn-ECF. (**D**) Bar graph showing the relative amplitude of zinc-mediated currents in cells expressing ASIC1a, ASIC2a or both. n = 5. * *p* < 0.05 vs. control, one-way ANOVA followed by post hoc Dunnett’s test.

**Figure 4:**
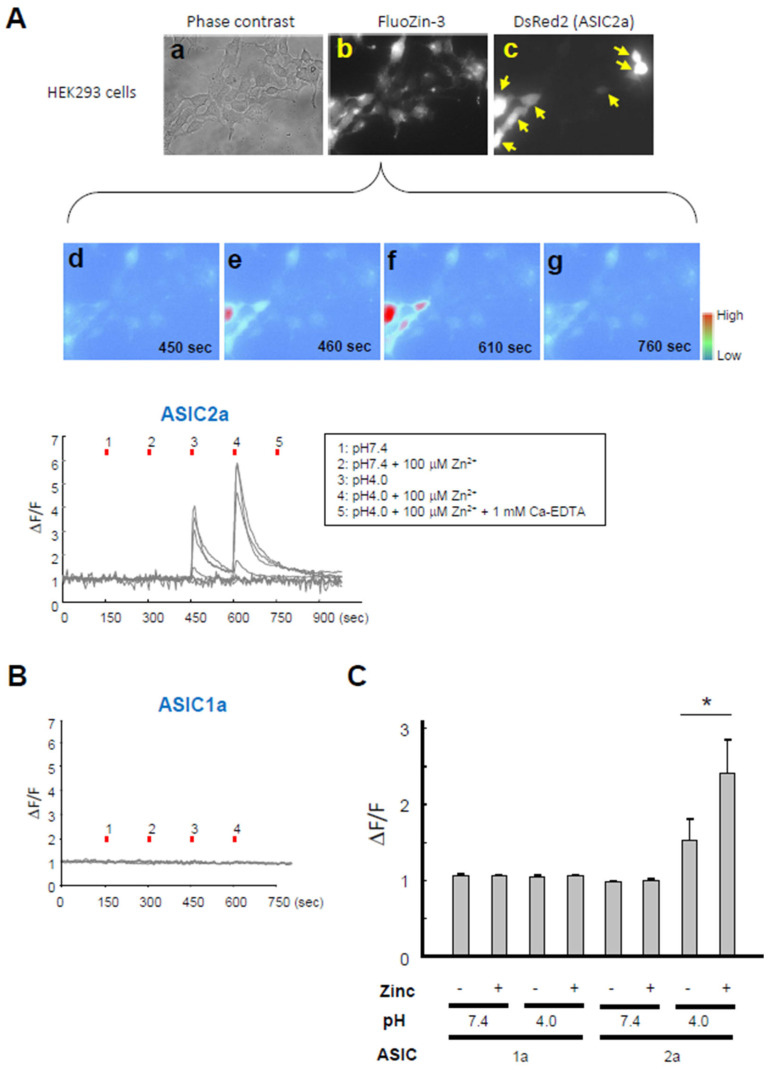
ASIC2a-dependent acid-induced zinc increases in HEK293 cells. HEK293 cells were transfected with ASIC2a-DsRed2 (**A**) or ASIC1a-DsRed2 (**B**). After 2–3 days, cells were incubated with FluoZin-3. (**A**) Representative phase-contrast (**a**) and FluoZin-3 fluorescence (**b**) are shown. ASIC2a-transfected cells were identified by the expression of DsRed2 (indicated with yellow arrows) (**c**). (**d**–**g**) Example images showing time-dependent changes in FluoZin-3 fluorescence. Relative changes in FluoZin-3 fluorescence when indicated solutions were perfused for 10 s to the cells expressing ASIC2a. (**B**) Relative changes in FluoZin-3 fluorescence when indicated solutions were perfused to the cells expressing ASIC1a. (**C**) Bar graph showing relative changes in peak zinc fluorescence intensities in response to perfusions of ECF with different pH values indicated. n = 15–16 cells from three independent experiments. * *p* < 0.05 vs. pH 4.0 with zinc, Fisher’s LSD test.

Data are presented as means ± SEM. Kaleidagraph 4.1 (Synergy Software, Reading, PA, USA) or SigmaPlot 10 (Systat Software, Palo Alto, CA, USA) was used for statistical analysis. For normally distributed data, the differences between the groups were compared using an unpaired Student’s *t*-test. One-way ANOVA followed by Dunnett’s post hoc test (Figures 1–3) and Fisher’s least significant difference (LSD) test (Figures 4 and 6) was used to compensate for multiple experimental procedures. A *p* value < 0.05 was considered statistically significant.

## 3. Results

### 3.1. Zinc Administration Increases Acid-Induced Currents in PcTx1-Insensitive Neurons

ASIC1a, ASIC2a, and ASIC2b are commonly expressed in neuronal cells. ASIC1a, the most common subunit, forms homotrimer or heterotrimer channels such as homomeric ASIC1a and heteromeric ASIC1a/2b, which are sensitive to an inhibitor PcTx1 [22,23,24], which binds to extracellular cysteine-rich domains of ASIC1a and inhibits the channel by increasing its apparent H^+^ affinity [25]. We first examined the effect of zinc on ASIC currents in primary cultured mouse cortical neurons. Acid application generated large inward currents in all neurons (Figure 1). We divided neurons into two types according to the sensitivity of ASIC currents to PcTx1 inhibition. In type 1 neurons (16 out of 39 cells, ~41%), which are less sensitive to PcTx1 (the amplitude of the current was reduced by less than 10%), application of zinc (0.1 mM) to normal ECF potentiated ASIC currents (Figure 1A,B). The lack of sensitivity of ASIC currents triggered by pH 6.0 to PcTx1 in type 1 neurons suggested that the currents are primarily mediated by heteromeric ASIC1a/2a channels. On the other hand, in type 2 neurons (23 out of 39 cells, ~59%), which are sensitive to PcTx1 inhibition, addition of zinc (0.1 mM) to ECF had no effect on the amplitude of ASIC currents (Figure 1B,D). The currents in these neurons are primarily mediated by homomeric ASIC1a and/or heteromeric ASIC1a/2b channels.

### 3.2. Acid Activates Zinc-Mediated Currents Which Are Inhibited by Amiloride

Potentiation of ASIC currents in type 1 neurons by an addition of zinc to the normal ECF suggested two possibilities: zinc enhances the currents mediated by other cations (Na^+^ and/or Ca^2+^), as suggested by the studies of Baron et al. [26], or zinc itself permeates through ASICs. To test the later possibility, Na^+^, K^+^, Ca^2+^, and Mg^2+^ in the normal ECF were substituted by N-methyl-D-glucamine (NMDG), a non-permeable cation, and 0.3 mM Zn^2+^ (Zn-ECF). As a result, lowering pH from 7.4 to 5.0 clearly induced an inward current, although the amplitude of the current was much smaller than that activated in normal ECF (Figure 2A). As expected for homomeric ASIC2a and/or heteromeric ASIC1a/2a channels, acid-induced currents in these neurons were inhibited by amiloride, a non-specific ASIC inhibitor, but not PcTx1, which only inhibits homomeric ASIC1a and heteromeric ASIC1a/2b channels (Figure 2). These findings suggested that homomeric ASIC2a and/or heteromeric ASIC1a/2a channels are likely zinc-permeable.

### 3.3. ASIC2a-Containing Channels Are Zinc-Permeable

To provide more evidence that ASIC2a-containing channels are zinc-permeable, we performed experiments in CHO cells expressing different subunits of ASICs. CHO cells are often used for examining ASIC currents because of no endogenous pH-sensitive current [27,28]. CHO cells were transfected with ASIC1a, ASIC2a, or both, followed with patch-clamp recordings. First, cells were perfused with normal ECF, and acid-generated currents were recorded. After recording stable ASIC currents, normal ECF was replaced with Zn-ECF. As shown in Figure 3, lowering pH from 7.4 to 5.0 in normal ECF activated large inward currents in CHO cells expressing ASIC1a, ASIC2a, or ASIC1a/2a channels. However, when normal ECF was replaced with Zn-ECF, lowering pH to 5.0 generated a small but clear inward current in CHO cells expressing ASIC2a or ASIC1a/2a, but not ASIC1a channels. These results provided additional evidence that ASIC2a-containing channels conduct zinc.

### 3.4. Activation of ASICs Increases Intracellular Zinc Concentration in an ASIC2a-Dependent Manner

To provide direct evidence that activation of ASIC2a-containing ASICs induces increases in intracellular zinc concentration, we performed fluorescence zinc imaging in HEK293 cells transfected with ASIC2a and loaded with FluoZin-3, a zinc specific fluorescence indicator [29]. ASIC2a-transfected cells were identified with the fluorescence protein, DsRed2 (red), and FluoZin-3 fluorescence signals (green) were measured (Figure 4A). While zinc application alone without changing pH did not alter intracellular zinc levels, application of acidic ECF (pH 4.0) increased the signal intensities in the absence of added extracellular zinc (Figure 4A). Addition of 100 μM zinc facilitated the acid-induced increase in intracellular zinc signal (Figure 4A,C). However, the acid-induced increase in intracellular zinc signal was not observed in the presence of 1 mM Ca-EDTA, a zinc chelator. Similar experiments were performed in cells transfected with ASIC1a, but neither application of acidic ECF nor addition of zinc altered the fluorescence intensities (Figure 4B,C). Thus, acid-induced increase in intracellular zinc depends on the presence of ASIC2a, further supporting that ASIC2a-containing channels are zinc-permeable.

### 3.5. Activation of ASICs Increases Intracellular Zinc Concentration in Cultured Mouse Cortical Neurons

**Figure 5:**
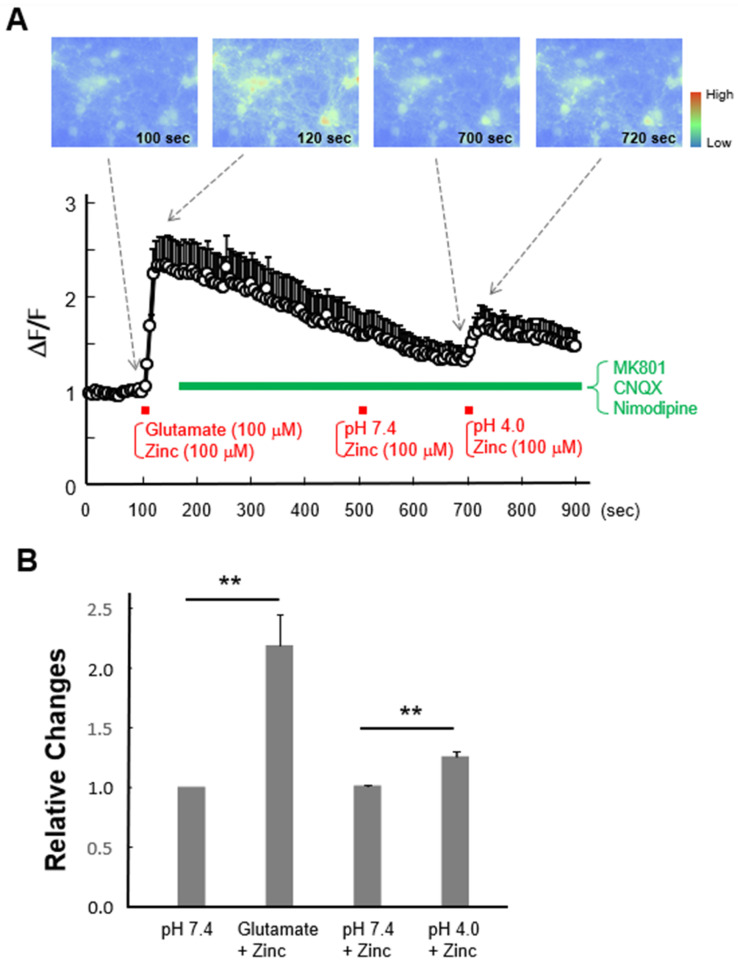
Acid-induced zinc increases in primary cultured mouse cortical neurons. Representative images and traces showing time-dependent changes in FluoZin-3 fluorescence in cultured mouse cortical neurons in response to perfusion of different solutions indicated. Similar trends were observed from three independent experiments. (**A**) The trace represents an average fluorescence intensity from seven randomly selected cells in an experiment. (**B**) Relative changes in peak intensities of FluoZin-3 fluorescence compared to ones immediately before indicated solution changes shown in (**A**). ** *p* < 0.01 vs. pH 7.4 or pH 7.4 + Zinc, Student’s *t*-test.

Next, we examined whether activation of ASICs induces intracellular zinc increase in neuronal cells. Primary cultured mouse cortical neurons were loaded with FluoZin-3 followed with fluorescence zinc imaging. As a positive control, application of zinc with glutamate increased the fluorescence signals (Figure 5A,B), consistent with the previous findings that glutamate receptors are zinc-permeable [9] and activation of glutamate receptors induces zinc influx [8,10]. We then blocked glutamate receptors and VDCCs and observed whether activation of ASICs induces increase in intracellular zinc. Application of zinc with pH 7.4 ECF did not change the fluorescence signals, but application of zinc in acidic ECF (pH 4.0) clearly increased zinc fluorescence (Figure 5A,B).

### 3.6. Zinc Facilitates Neuronal Injury Under Severe Acidic Condition

**Figure 6:**
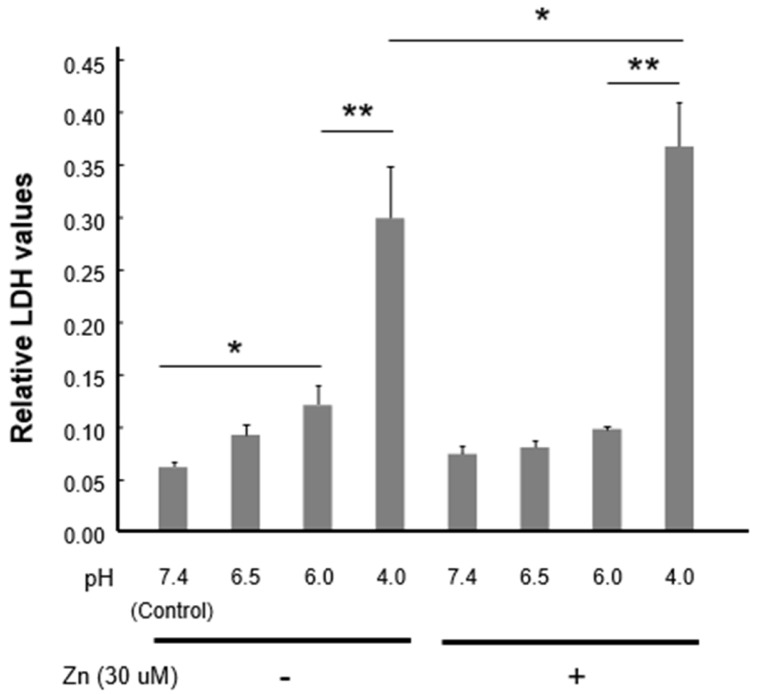
Zinc-enhanced cell injury under acidic conditions. Cultured mouse cortical neurons were incubated with ECF at different pH values for 1 h in the absence or presence of zinc. Cells were then incubated in normal growing media for 5 h. Relative LDH releases normalized to the maximal values (obtained by treating cells with Triton X-100, see Section 2.7) are shown. n = 6–12. * *p* < 0.05, ** *p* < 0.01 vs. the indicated treatments, Fisher’s LSD test.

Finally, we examined whether zinc influx induced by acidic condition influences neuronal injury. Primary cultured mouse cortical neurons were treated with ECF containing no or 30 µM zinc at various pH levels in the presence of inhibitors for glutamate receptors and VDCCs for 1 h. They were then incubated with normal culture media for 5 h, followed by the measurement of lactate dehydrogenase (LDH) release. Zinc administration (30 µM) did not significantly influence the baseline LDH release at pH7.4 and pH6.5. Interestingly, zinc application appears to attenuate acid-induced LDH release at pH6.0, likely through its inhibition of ASIC1a-containing channels, as reported in our previous studies [20]. However, zinc application significantly enhanced the LDH release at pH 4.0 (Figure 6). Taken together, these findings suggest that in severe acidic conditions where ASIC2a-containing channels are activated [30], zinc entry through these channels likely acerbates acidotoxicity.

## 4. Discussion

Zinc is involved in a range of essential biological processes, including immune function, maintenance of taste, growth, development, and general healing [31,32,33]. Zinc deficiency can lead to various health issues such as dermatitis, taste disorders, and growth impairments [33]. Conversely, excessive zinc, such as from supplemental overdoses or industrial exposure to zinc fumes, can also cause harmful effects [32,33]. Additionally, pathophysiological events like ischemia–reperfusion can elevate zinc levels in neural tissues, contributing to zinc toxicity in neurons [33,34].

Physiological zinc levels in the brain vary across different compartments. Extracellular concentrations of Zn^2+^ typically range from 10 to 100 nM but can transiently increase during synaptic activity [35,36]. Upon synaptic release, Zn^2+^ concentrations can reach 100–300 µM, particularly under pathological conditions [37]. Therefore, the use of 300 µM Zn^2+^ in the present study is physiologically or pathologically relevant. Intracellular Zn^2+^ concentrations are tightly regulated, typically between 0.1 and 5 nM, but can rise to 1–10 µM under pathological conditions such as ischemia [35,36]. While synaptic Zn^2+^ levels can reach hundreds of micromolar, direct measurements of intracellular Zn^2+^ during synaptic transmission remain challenging.

Zinc is known to accumulate in synaptic vesicles at the presynaptic terminals, and ischemia–reperfusion-induced depolarization triggers the release of zinc into synaptic clefts. This vesicular accumulation is largely dependent on the zinc transporter ZnT3, and its deficiency leads to decreased vesicular zinc, which is associated with cognitive disorders like dementia [38,39,40]. Thus, maintaining normal extracellular zinc levels in synaptic clefts is essential for proper neuronal function.

Excessive activation of glutamate receptors and intracellular calcium overload in postsynaptic neurons have been well-established as key contributors to neuronal injury during brain insults such as cerebral ischemia [3,4]. However, it has also become clear that zinc entry, via various receptors and channels such as glutamate receptors, VDCCs, and TRPM7, plays an additional role in neuronal injury [7,12]. It is worth mentioning that although glutamate receptors and voltage-gated calcium channels are known to be Ca^2+^ and zinc-permeable, most of these channels are inhibited by acidosis. In the present study, we found that the activation of ASIC2a-containing channels induces zinc influx and zinc addition exacerbates severe acidosis-induced neuronal injury. Since extracellular acidosis is a prominent feature in brain ischemia and activation of ASICs plays a critical role in ischemic brain injury [14], our data suggest that targeting these channels may help mitigate zinc-mediated neurotoxicity in brain ischemia. 

Zinc is a trace element, and many physiological solutions used in various studies already contain non-negligible zinc concentrations [41,42,43,44], which may complicate the ability to detect clear effects of added zinc. More commonly, effects are observed via zinc chelation [45], and the role of zinc in ischemia–reperfusion injury has been further recognized when chelators such as TPEN or Ca-EDTA, which bind zinc more effectively than Ca^2+^, inhibit neuronal damage [5,6].

There are two main types of zinc transporters, ZnTs and ZIPs, that regulate zinc homeostasis [46,47]. Zinc-permeable ion channels, such as ASICs, likely operate in localized regions, such as synaptic terminals, and their activation can lead to rapid zinc influx, causing an immediate rise in intracellular zinc concentrations. This rapid elevation may serve a physiological signaling function, but an overload can result in zinc toxicity.

As noted earlier, ion channels and receptors like VDCCs, NMDA receptors, and TRPM7 are known to be permeable to both calcium and zinc [7,8,9,10,48]. Most of these channels are expressed in neuronal cells, and zinc is co-released with glutamate from synaptic vesicles, increasing zinc concentration at synaptic sites during neurotransmission. Activation of zinc-permeable channels at postsynaptic terminals raises intracellular zinc levels, contributing to physiological zinc-dependent functions. However, during pathological conditions such as brain ischemia, excessive zinc entry via these channels can also exacerbate brain injury [49]. Despite these findings, therapeutic targeting of these channels has not yet resulted in satisfactory neuroprotection for a variety of reasons [50]. There are limitations in our studies. First, acidic conditions used in our toxicity assay (e.g., pH 4.0) did not simulate a normal in vivo ischemic condition. Second, zinc-permeable channels other than ASIC2a were not studied comparatively. Future studies will explore acid-mediated zinc toxicity in more physiologically/pathologically relevant conditions and compare the relative contribution of ASIC-mediated toxicity with other known zinc-permeable channels.

Previously, we demonstrated that activation of ASIC1a channels is central to is-chemic brain injury, with calcium influx partially contributing to neuronal damage [14,15]. The role of ASIC2a in ischemic injury is less established, although we have shown that ASIC2a plays a region-specific role in acidosis- and ischemia-induced neuronal injury [51]. Our findings suggest that ASIC2a facilitates ASIC1a surface trafficking, and deletion of ASIC2a reduces acid-activated current in cortical and striatal neurons. Importantly, ASIC2a deletion also protected the brain from ischemic damage [51]. However, as our data supporting the role of ASIC2a-containing channels-mediated zinc influx in ischemic injury is limited, further research is required to determine whether this mechanism contributes significantly to ischemic brain injury, in addition to its role in regulating ASIC1a expression and function.

A recent study by Lai et al. revealed that glutamate potentiates ASIC1a activity through a direct binding [52]. This finding suggests that glutamate released during ischemia/reperfusion enhances ASIC1a activity, indicating that ASIC overactivation plays a critical role in neuronal injury under acidic conditions. This discovery further highlights the importance of ASICs in ischemic brain injury and could explain why blocking glutamate receptors alone is ineffective in stroke interventions in humans. Given the widespread expression of ASIC1a and ASIC2a in brain neurons, and the ability of homomeric ASIC1a to conduct calcium and heteromeric ASIC1a/2a to conduct zinc, acidosis-induced activation of these channels is expected to cause surges of calcium and zinc in ASIC1a- and ASIC1a/2a-dominant neurons, respectively. However, due to the limitations in the current study, further experiments, including those in vivo, are needed to conclusively demonstrate the role of zinc entry through ASIC2a-containing channels in ischemic neuronal injury and other neurological disorders.

## 5. Conclusions

In conclusion, our findings highlight the critical role of ASIC2a-containing channels in mediating zinc influx under acidic conditions. Unlike ASIC1a, which does not permit zinc entry, activation of ASIC2a-containing channels facilitates zinc influx, which exacerbates severe acidosis-induced neurotoxicity. These results suggest that targeting ASIC2a-containing channels might offer potential therapeutic strategies to mitigate acidosis-mediated neuronal damage. Further investigations into the precise mechanisms by which zinc influx through ASIC2a-containing channels contributes to neurotoxicity will be helpful in developing targeted interventions in ischemic brain injury.

## Data Availability

The main data supporting the results in this study are available within the paper. The raw and analyzed datasets generated during the study are available for research purposes from the corresponding author(s) on reasonable request.

## Abbreviations

The following abbreviations are used in this manuscript:

AMPA	α-amino-3-hydroxy-5-methyl-4-isoxazolepropionic acid
ASICs	Acid-sensing ion channels
CHO	Chinese Hamster Ovary
ECF	Extracellular solution
HEK293	Human Embryonic Kidney 293
LDH	Lactate dehydrogenase
LSD	Least significant difference
NMDA	N-methyl-D-aspartate
NMDG	N-methyl-D-glucamine
PBS	Phosphate-buffered saline
TRPM7	Transient receptor potential melastatin 7
VDCCs	Voltage-dependent calcium channels
Zn-ECF	ECF with zinc as the only charge carrier
